# Network of Palladium-Based Nanorings Synthesized by Liquid-Phase Reduction Using DMSO-H2O: In Situ Monitoring of Structure Formation and Drying Deformation by ASEM

**DOI:** 10.3390/ijms21093271

**Published:** 2020-05-05

**Authors:** Takuki Komenami, Akihiro Yoshimura, Yasunari Matsuno, Mari Sato, Chikara Sato

**Affiliations:** 1 Graduate School of Science and Engineering, Chiba University, Chiba 263-8522, Japan; komenamiftn@gmail.com (T.K.); a.yoshimura@chiba-u.jp (A.Y.); 2 Health and Medical Research Institute, National Institute of Advanced Industrial Science and Technology (AIST), Tsukuba 305-8566, Japan; ma-satou@aist.go.jp

**Keywords:** atmospheric scanning electron microscopy, critical point drying, nanoparticles, nanostructures, precious metals

## Abstract

We developed a liquid-phase synthesis method for Pd-based nanostructure, in which Pd dissolved in dimethyl sulfoxide (DMSO) solutions was precipitated using acid aqueous solution. In the development of the method, in situ monitoring using atmospheric scanning electron microscopy (ASEM) revealed that three-dimensional (3D) Pd-based nanonetworks were deformed to micrometer-size particles possibly by the surface tension of the solutions during the drying process. To avoid surface tension, critical point drying was employed to dry the Pd-based precipitates. By combining ASEM monitoring with critical point drying, the synthesis parameters were optimized, resulting in the formation of lacelike delicate nanonetworks using citric acid aqueous solutions. Precipitation using HCl acid aqueous solutions allowed formation of 500-nm diameter nanorings connected by nanowires. The 3D nanostructure formation was controllable and modifiable into various shapes using different concentrations of the Pd and Cl ions as the parameters.

## 1. Introduction

Precious metal nanomaterials, especially Au, Pt, and Pd, have attracted significant attention in a wide range of fields such as chemical catalysts, nanotechnology, and material chemistry owing to their unique electrical, catalytic, and optical properties [1,2]. Pd is well known for its ability to absorb hydrogen and is a promising material for hydrogen storage and gas sensing applications [3,4]. Pd also has excellent catalytic activity for organic synthesis and exhaust gas purification [5,6]. In these industrial fields, the large surface area and high catalytic performance of Pd nanomaterials are of major interest in improving the reaction efficiency and selectivity [5,7].

Over the past decade, significant progress toward the development of synthetic techniques for the fabrication of Pd nanostructured materials with controllable morphologies and dimensions has been made. Physical synthesis techniques, hydrothermal methods, electrochemical deposition, chemical reductions, etc. have been investigated for the synthesis of Pd and Pd-based nanomaterials [8].

As a kind of face-centered cubic (fcc) metal, Pd tends to form symmetric morphologies instead of nanowires or nanosheets. Therefore, in order to obtain the desirable framework, it is necessary to effectively control the thermodynamics and kinetics processes involving reduction and crystal growth [9].

**Figure 1:**
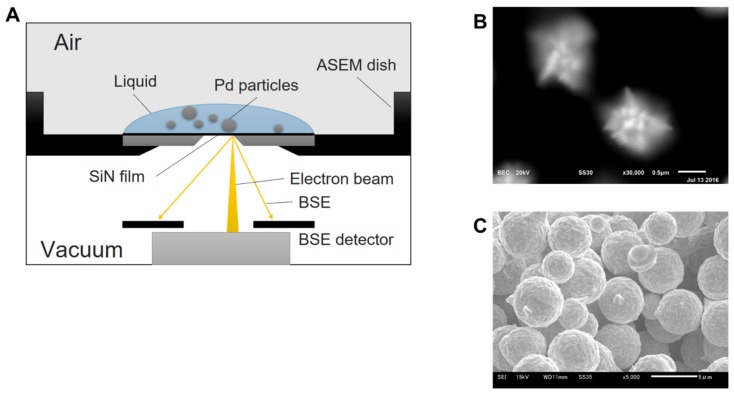
Images of Au micrometer-size particles taken by atmospheric scanning electron microscopy (ASEM) and a traditional SEM. (**A**) Schematic diagram of ASEM system. The ASEM system is composed of an inverted SEM system and a silicon nitride (SiN) thin-film electron transparent windowed sample holder, and enables researchers to observe samples immersed in liquid under atmospheric pressure. Resolution of ASEM is 8 nm near SiN membrane [15]. (**B**) ASEM images of gold particles synthesized in the liquid after 37 min at a magnification of ×30,000 [11]. (**C**) Image of gold particles after filtered and dried, taken by a traditional SEM (11).

In our previous studies, a facile liquid-phase synthesis method was developed for the synthesis of Au micrometer-size particles [10]. The method involved leaching gold using a DMSO solution containing copper halide, followed by the selective precipitation of gold using acid aqueous solution. Furthermore, in situ monitoring of the synthesis in the liquid was successfully conducted using our developed atmospheric scanning electron microscopy (ASEM) in order to investigate the mechanism of particle formation [11]. The ASEM system is composed of an inverted SEM system and a silicon nitride (SiN) thin-film electron transparent windowed sample holder, and enables researchers to observe samples immersed in liquid under atmospheric pressure (Figure 1A) [12,13,14]. ASEM monitoring revealed that the Au precipitated with a confeito-like shape in DMSO–H2O solvent (Figure 1B) and was deformed after the drying process (Figure 1C). Therefore, the drying process may significantly affect the Pd nanostructure in liquid-phase synthesis.

Herein, we report a liquid-phase synthesis method of Pd-based nanostructure in which Pd dissolved in dimethyl sulfoxide (DMSO) solutions was precipitated using acid aqueous solution (Figure S1). Furthermore, in situ monitoring of the synthesis in the liquid was conducted using ASEM in order to investigate the particle formation as well as deformation during the drying process possibly due to the surface tension. To avoid surface tension, the delicate Pd nanostructures were dried using critical point drying, which replaces the solvent with supercritical CO_2_ fluid in order to evaporate liquid without significant surface tension. Using these methods, the synthesis method was further optimized for various three-dimensional (3D) nanostructure fabrications by modifying the Pd and Cl ion concentrations as the parameters.

## 2. Results

### 2.1. In situ Monitoring of Pd-Based Nanostructure Formation and Deformation

**Figure 2:**
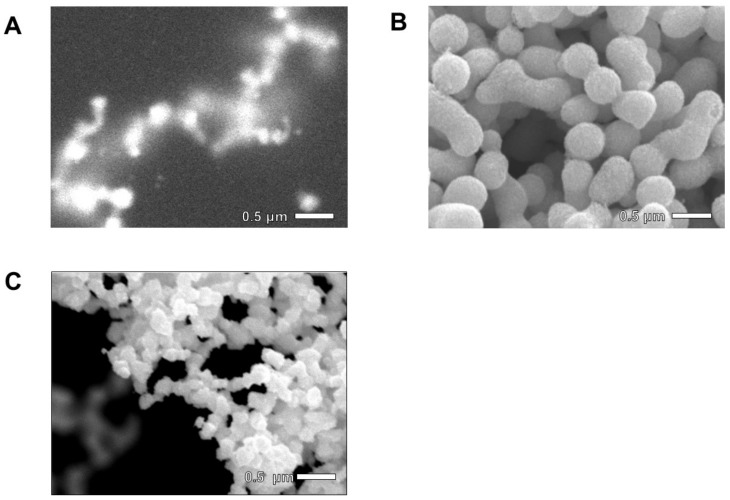
Images of 3D Pd-based nanostructures taken by ASEM and a traditional SEM. (**A**) ASEM image of Pd-based particles in liquid after 25 min of mixing. In the liquid phase, Pd-based particles formed lacelike network nanostructures. (**B**) Image of Pd-based particles after drying at 383 K for 24 h, taken by traditional SEM. Dried particles formed spherical shapes of micrometer size with smooth surfaces. (**C**) Image of Pd-based particles after critical point drying process taken by a traditional SEM. It is clearly shown that dried particles formed lacelike network nanostructures.

To investigate the mechanism of Pd-based nanostructure formation, liquid-phase observation was conducted under monitoring using ASEM. Pd dissolved in DMSO was mixed with 2.0 M citric acid aqueous at a volume ratio of 1:3, and the reaction mixture was monitored (Supplementary Video S1, in which the white bar shows 5 µm.). ASEM monitoring showed that Pd-based particles synthesized in the reaction were connected and formed lacelike networks (Figure 2A). In ASEM monitoring, the connections far from the SiN film were blurred because the electron beam of ASEM broadens as the distance from the SiN film increases [15,16]. From the image in Figure 2A, it appears that several dozen or more Pd-based particles are connected together to form a lacelike 3D cluster. Figure 2B shows an image of the particles dried at 383 K for 24 h, taken by a traditional SEM. Unlike the structure immersed in the liquid, the dried particles were spherical, had smooth surfaces, and were sometimes connected. These results suggest that a 3D Pd-based nanostructure formed in the liquid was deformed into micrometer-size spherical particles during the drying process.

To investigate the mechanism of the deformation of the Pd-based nanonetwork, in situ monitoring at the moment of drying was carried out. The in situ videos are shown in Supplementary Video S2, in which the white bar shows 1 µm. At the moment of drying, ASEM imaging showed that the Pd-based nanostructure synthesized in the liquid (Figure 2A) aggregated to micrometer-sized particles. It is suspected that the deformation of the 3D structure can be attributed to the surface tension in the solvent.

### 2.2. Production of 3D Nanostructure

To avoid surface tension in the drying, critical point drying was used to dry the delicate Pd nanostructures. The advantage of using supercritical CO_2_ in the drying process compared to normal drying has been recognized as eliminating the surface tension forces created by the rinse fluid [17]. Figure 2C shows a higher-magnification SEM image of the 50–100 nm-size Pd-based particles connect to form 3D networks obtained by mixing Pd-dissolved DMSO with 2.0 M citric acid aqueous solution in a volume ratio of 1:3, followed by critical point drying. The shape and size of the structure obtained by critical point drying correspond reasonably well to the structure in the liquid (Figure 2A) and different from those obtained by the conventional drying (Figure 2B). Thus, it is confirmed that the critical point drying successfully removed the solution without significant deformation of the particles.

**Figure 3:**
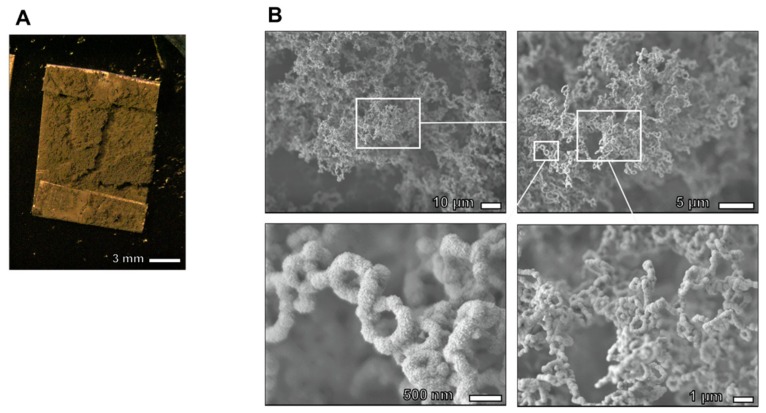
Fabrication of 3D nanostructure using critical point drying method. (**A**) Optical microscope image and FE-SEM images of nanoring-chained 3D structure obtained by adding HCl aqueous. The precipitates deposited on Pd plate at a thickness of 100 µm or more. (**B**) SEM images of Pd 3D nanostructure. Nanoring structures with diameters of approximately 500 nm were connected to each other to form 3D chain structure.

A different type of nanostructure was constructed on a Pd plate when the Pd-dissolved DMSO was mixed with 0.1 M HCl aqueous solutions. The precipitates were deposited on the Pd plate at a thickness of 100 µm or more (Figure 3A). SEM images in Figure 3B show that the precipitates obtained under this condition formed nanoring structures with a diameter of approximately 500 nm that were connected to each other to form 3D chain networks. The thickness of each nanoring was approximately 200–300 nm.

**Figure 4:**
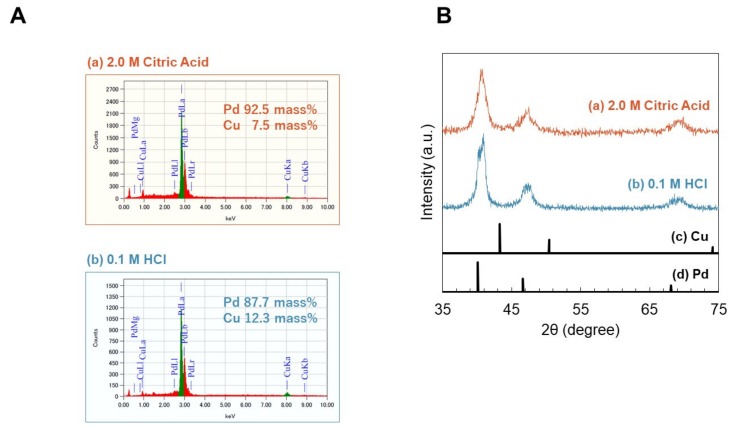
Characterization of 3D nanostructure. (**A**) The weight composition ratio of the precipitates obtained by the addition of citric acid aqueous and HCl aqueous. The composition of the precipitates were determined by Energy Dispersive X-ray Spectrometry (EDS) analysis, and only Cu and Pd were detected. (**B**) Diffraction patterns of precipitates obtained by addition of 2.0 M citric acid aqueous and 0.1 M HCl aqueous.

A composition analysis conducted by SEM-EDS shows the weight composition ratio of the precipitates obtained by the addition of citric acid aqueous and HCl aqueous (Figure 4A). Pd and Cu were detected in both precipitates, and no other elements were detected. Figure 4B shows the powder X-ray diffraction pattern of the precipitates and the typical diffraction patterns [18] of Cu metal and Pd metal. The crystal structures of Cu metal and Pd metal are cubic phases and Cu has a smaller lattice spacing. The peaks of both precipitates are slightly shifted to the wide-angle side from the peak of Pd metal and are not split. This result indicates that the precipitates obtained in this experiment are solid solutions of Pd and Cu. The mean crystalline sizes of the precipitates were estimated using on Scherrer’s equation, in which shape factor, *K*, was set as 0.9. This resulted in the mean crystalline sized of 5.1 nm and 6.0 nm for the precipitates obtained by the addition of citric acid aqueous and HCl aqueous, respectively. This indicates that primary particles with a diameter of 5–6 nm were first synthesized in the reaction, and then formed the nanoring structures, which is consistent with SEM observation with higher resolution (Figure S2).

**Figure 5:**
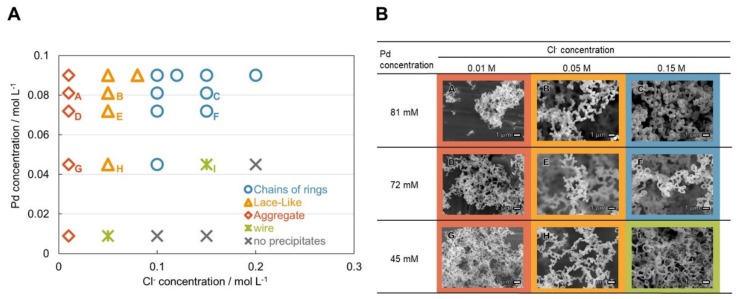
Shape control of the nanostructure by Pd and Cl concentration. (**A**) Diagram of nanostructure shapes as function of Pd and Cl concentrations. (**B**) SEM images of precipitates formed at various Pd and Cl concentrations. Pd particles produced a lacelike 3D network (B, E, H) at a Cl concentration of 0.05 M, and produced chains of nanoring structures and nanowires (C, F, I) at a Cl concentration of 0.15 M. The size of the nanoparticles and thickness of the rings decreased as the Pd concentration decreased.

Because it is known that the dispersion stability of particles in solution depends on the ionic strength, the synthesis of Pd-based nanostructures was carried out under various Cl ion concentrations of HCl aqueous solution and Pd concentrations of DMSO. The precipitate obtained under each condition was dried with a critical point dryer, and its shape was confirmed with SEM. Figure 5A shows a diagram of the shapes of the nanostructures as a function of the Pd and Cl ion concentrations. The horizontal axis is the concentration of Cl ions in the HCl aqueous, and the vertical axis is the concentration of dissolved Pd in DMSO before mixing. SEM images of the nanostructures are shown in Figure 5B. The characters in the images correspond to the alphabet-tagged conditions in Figure 5A. By controlling the Pd and Cl ion concentrations, aggregates, lacelike structures, chains of nanoring structures, and nanowire structures were obtained. From a comparison of SEM images in Figure 5B, it was found that the micrometer-size structures such as the lacelike shapes and chains of nanoring structures depend on the Cl ion concentration. On the other hand, with a decrease in the Pd concentration, the sizes of the nanoparticles making up the 3D structure and the thickness of the nanoring structures became smaller. Therefore, as a formation mechanism of the structure, it appears that primary particles (having a size dependency on the Pd ion concentration) precipitate first and then link together to form a 3D structure having a shape dependent on the Cl ion concentration. In addition, no precipitate was obtained under conditions of high Cl ion concentration and low Pd ion concentration. The reason is assumed to be because Pd ions were stabilized in the solution by forming Pd^2+^–Cl–DMSO complex [19,20] such as [PdCl_4_] ^2−^, [PdCl_3_(DMSO)]^−^, and [PdCl_2_(DMSO)_2_] owing to high concentrations of Cl ions and DMSO for Pd.

## 3. Discussion

As it is shown in Figure S1, Cu^2+^ in DMSO acts as a strong oxidant that dissolves Pd, which consists of the following anodic and cathodic half-cell reactions.

(1) $$\mathrm{Pd}\;\to {\mathrm{Pd}}^{2+}+2e^{-}$$

(2) $${\mathrm{Cu}}^{2+}+e^{-}\;\to {\mathrm{Cu}}^{+}$$

Actually Pd^2+^, Cu^2+^ and Cu^+^ form complexes with Cl^−^ in DMSO [10,19,20], however, for simplicity, they are shown as Pd^2+^, Cu^2+^ and Cu^+^. The increase in the concentration of Cl^−^ in DMSO, will enhance the dissolution of Pd. The addition of water to the DMSO solutions will drastically change the characteristic of the solution, resulting in that the redox potential of (1) becomes more positive than that of (2). Therefore, the reverse reactions take place, which precipitate Pd.

Assuming a mechanism in which primary particles link together to form 3D structures, a change in the shape of the 3D structure with the Cl ion concentration is considered a change in the stability of the colloidal dispersion. According to the DLVO theory [21,22], the colloidal stability of charged particles increases with an increase in the ion strength in solutions because of the higher energy potential barrier between particles [23,24]. On the other hand, another particle interaction occurs in the mixed solvent [25,26,27]. In general, since the colloid surface strongly adsorbs either component, the solvent composition around the colloid becomes nonuniform. When such colloidal particles get close to each other, nonuniform layers overlap, so that an interaction occurs between the particles. Beysens and Esteve reported that such interaction behaves as an attractive force, and the colloid particles aggregate [28]. In addition, DMSO molecules strongly adsorb to the surface of Pd nanoparticles in pure DMSO solvent [29]. Therefore, it is assumed that not only the function of the Cl ion concentration but also the function of DMSO molecules plays an important role in the formation of 3D nanostructures.

## 4. Materials and Methods

### 4.1. Materials

Pd wire (99.95%, 0.2 mm diameter) and Pd plate (99.5%, 0.05 mm thickness) were purchased from Nilaco Corporation, Tokyo, Japan. NaCl (99%), DMSO (99%), HCl (35%–37%), Ethanol (99.5%), and CuCl_2_ (98%) were purchased from Wako Pure Chemical Industries, Ltd., Tokyo, Japan.

### 4.2. Pd Dissolution and Deposition

To 100 mL of DMSO, 20 mmol of CuCl_2_ and NaCl were added. Then, 10 mmol of Pd wire (99.95%, 0.2 mm diameter) was added to the DMSO solution and shaken at 357 K for approximately 8 h to dissolve the Pd until saturation. After the dissolution, the solution was filtered to remove the residue.

To precipitate Pd, 10 mL of Pd-dissolved DMSO was mixed with 30 mL of 2.0 M citric acid aqueous in a 50 mL centrifuge tube, shaken quickly and left at room temperature for 24 h. In the case of using HCl aqueous solution, a Pd plate was placed in the mixed solvent as a carrier because the precipitates formed a film on the surface of the sample tube. Specifically, 1 mL of Pd-dissolved DMSO, 3 mL of 0.1–2.0 M HCl acid aqueous, and a Pd plate (99.95%, 0.05 × 5 × 10 mm) was added in a 5-mL polypropylene tube, shaken quickly, and left at room temperature for 24 h.

For the shape control of the nanostructure by Pd and Cl concentration, Pd-dissolved DMSO was mixed with pure DMSO so that the volume ratio of Pd-dissolved DMSO: DMSO = 10:0, 9:1, 8:2, 5:5, 1.9, respectively.

### 4.3. ASEM Monitoring

The dynamic precipitation process in the liquid was live-imaged using the ClairScope™ ASEM system (JASM-6200; JEOL Ltd., Tokyo, Japan). For this, 40 µL of Pd-dissolved DMSO was dropped on the ASEM dish with a pipette, and the focus of the ASEM was adjusted. Next, the DMSO solution on the dish was collected in an Eppendorf tube with a pipette and mixed with 120 µL of 2.0-M citric acid aqueous solution. Subsequently, the solution was quickly returned to the dish and monitored by ASEM. The accelerating voltage of the ASEM was 20 kV, and backscattered electrons were recorded by a backscattered electron imaging detector.

For in situ monitoring of the deformation of the Pd nanonetwork at the moment of drying, a mixture of the sedimentation was placed on a SiN film window of the ASEM dish. The supernatant was replaced with pure water before monitoring because DMSO is a nonvolatile solvent. The solvent of the sedimentation solution was replaced with DDW four times; 10 µL of the solution was quickly removed and 10 µL of fresh DDW was dropped onto the ASEM dish. The ASEM monitoring was started immediately and carried out until the sample was completely dried under atmospheric conditions. The accelerating voltage of the ASEM was 20 kV, and backscattered electrons were recorded. The video was recorded at a frame rate of 2 frames/s.

### 4.4. Critical Point Drying

The prepared sedimentation solution was exchanged with ethanol four times to match the miscibility of liquid CO_2_. The ethanol-exchanged precipitate was loaded in a critical point dryer (EM CPD300; Leica Microsystems GmbH, Wetzlar, Germany), and then the drying chamber was filled with liquid CO_2_ by stirring. The solvent in the drying chamber was replaced with liquefied CO_2_ 20 times over approximately 30 min. Finally, the interior of the chamber was heated and pressurized to 313 K and 74 atm to make liquid CO_2_ into supercritical fluid, and then decompressed to atmospheric pressure over approximately 30 min to obtain dried samples. As a comparative study, the sedimentation solution was filtered and dried at 383 K for 24 h.

### 4.5. Characterization

The dried Pd-based nanostructure was observed using a scanning electron microscope (JSM-6510A, JEOL) and field-emission scanning electron microscope (JSM-7400F, JEOL). The accelerating voltages of the SEM and FE-SEM observations were 15 kV and 1.5 kV, respectively. The composition analysis of the precipitates obtained by the addition of 2.0 M citric acid aqueous solution and the addition of 0.1 M HCl acid aqueous solution was conducted by an EDS (EDAX Genesis; AMETEK Inc., Berwyn, PA, USA) mounted on the FE-SEM at an accelerating voltage of 15 kV. Powder X-ray diffraction patterns were measured using a D8 Advance (Bruker Corporation, Billerica, MA, USA) employing Cu-Kα radiation.

## 5. Conclusions

In situ monitoring using ASEM was conducted to investigate the mechanism of Pd-based particle formation. The results revealed that 3D Pd-based nanonetworks formed in the liquid were deformed to micrometer-size particles during the drying process. Because one of the reasons for this deformation could be surface tension of the solution, critical point drying was used to avoid the effect of surface tension and dry the 3D Pd-based nanostructure without significant deformation. As a result, we obtained the following 3D nanostructures: lacelike networks formed by the addition of citric acid aqueous and chains of nanoring structures by HCl acid aqueous. The shapes of the 3D nanostructure obtained by addition of HCl aqueous were controllable to lacelike structures, chains of nanoring structures, and nanowires by changing the concentrations of Pd and Cl ions. In this study, we synthesized complicated shapes such as nanoring structures and nanowires rapidly with the simple operation of solvent mixing. This method is categorized as a liquid-phase reduction method, one of the easiest production methods for 3D nanostructures.

## Supplementary Materials

Supplementary materials can be found at https://www.mdpi.com/1422-0067/21/9/3271/s1.

## Abbreviations

ASEM	Atmospheric scanning electron microscopy
3D	3-dimensional
DMSO	Dimethyl sulfoxide
