# Supplementary figures and images for: Network of Palladium-Based Nanorings Synthesized by Liquid-Phase Reduction Using DMSO-H2O: In Situ Monitoring of Structure Formation and Drying Deformation by ASEM
Source: Int J Mol Sci. 2020 May 5;21(9):3271. doi: 10.3390/ijms21093271 (PMC7247573; doi:10.3390/ijms21093271)

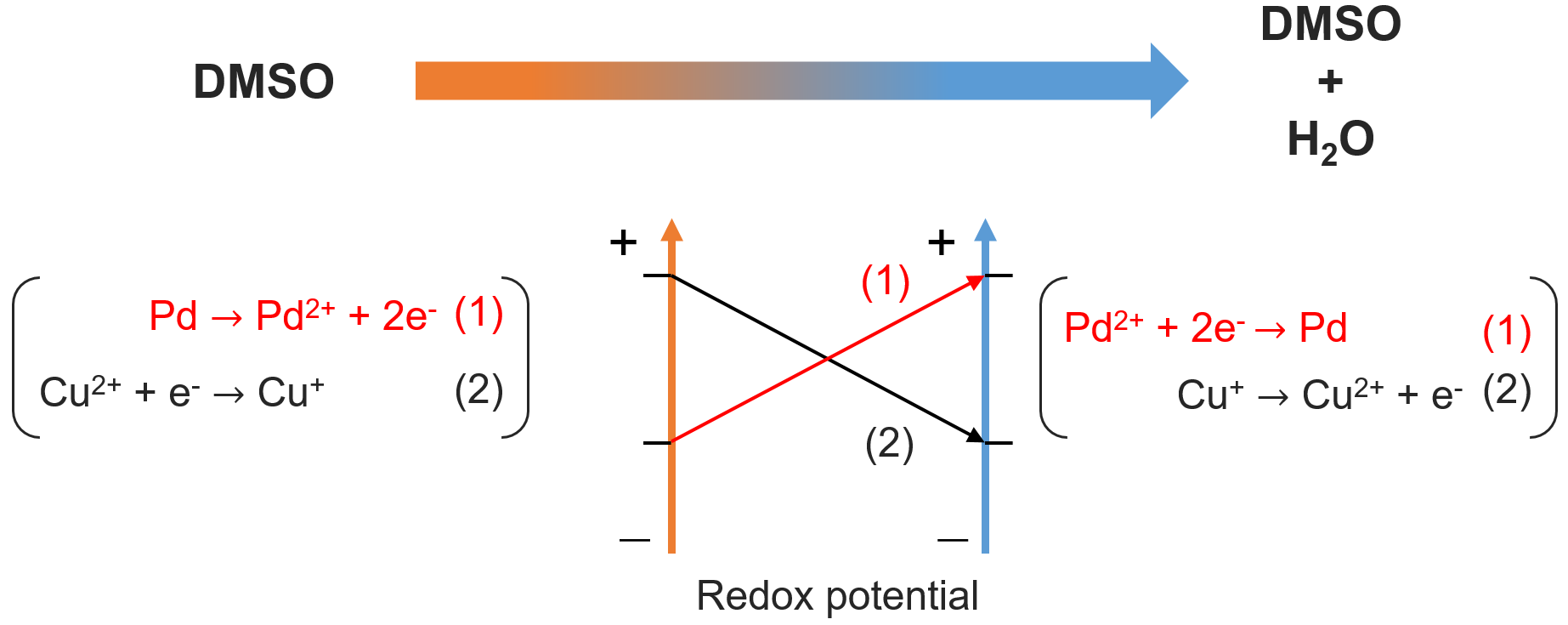

Supplement: Supplementary file 1 [file ijms-21-03271-s001.zip › Supplemental_figure1.tif]

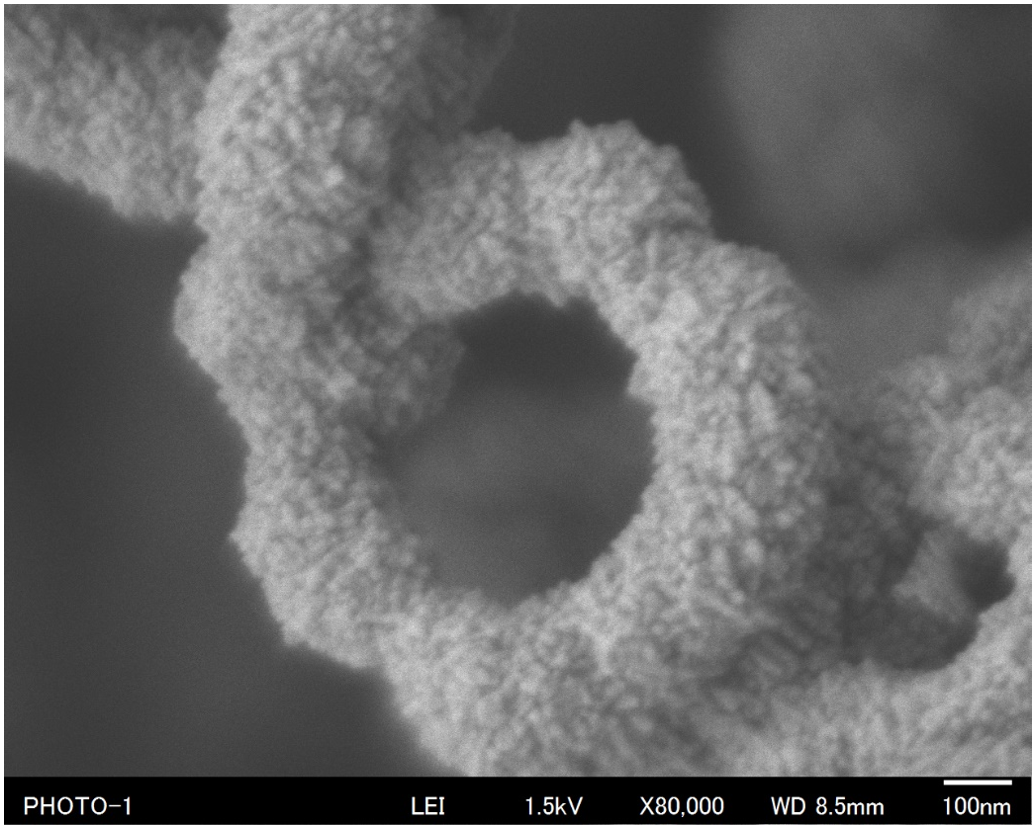

Supplement: Supplementary file 1 [file ijms-21-03271-s001.zip › Supplemental_figure2.tif]
